# Role of positron emission tomography-computed tomography in staging and early chemotherapy response evaluation in children with neuroblastoma

**DOI:** 10.4103/0972-3919.78249

**Published:** 2010

**Authors:** Madhavi Chawla, Rakesh Kumar, Sandeep Agarwala, Sameer Bakhshi, Devendra Kumar Gupta, Arun Malhotra

**Affiliations:** Department of Nuclear Medicine, All India Institute of Medical Sciences, New Delhi, India; 1 Department of Pediatric Surgery, All India Institute of Medical Sciences, New Delhi, India; 2 Department of Medical Oncology, All India Institute of Medical Sciences, New Delhi, India

**Keywords:** Neuroblastoma, neoadjuvant chemotherapy, PET-CT, staging, treatment response evaluation

## Abstract

**Background:**

To evaluate the role of positron emission tomography-computed tomography (PET-CT) in staging and determining early treatment response to chemotherapy in children with neuroblastoma (NB) and its correlation with the final outcome.

**Patients and Methods:**

Seventeen patients of NB with mean age of 51.5 months (age range 2-132 months; 14 males, 3 females) underwent serial 18F-flourodeoxygl ucose (FDG) PET-CT imaging. All 17 patients were for staging before any treatment. Twelve of 17 patients underwent I-131 meta-iodobezylguanidine (MIBG) scan and bone scan. MIBG uptake was seen in the primary lesion in 11/12 patients. MIBG uptake in bones was seen in 3/12 patients. All bone lesions were concordant on MIBG and bone scan. Early response to chemotherapy was evaluated after two cycles using PET-CT. A 30% reduction in longest diameter was taken as cut-off value for response on CT based on the response evaluation criteria in solid tumors criteria. Response on PET-CT was assessed using percentage improvement in lesion to background SUV ratio, taking a value of 50% as cut-off. Final outcome based on follow-up ranging from 6 to 43 months (mean 18.8 months) served as reference.

**Results:**

All 17 patients showed increased FDG uptake at the primary site. Seven of the 17 patients (41.2%) showed metastasis. Lymph nodes were the most common site of metastatic disease followed by bone, bone marrow, lung and meninges. For response evaluation, change in the size of the primary tumor was noted in 11/17 (64.7%) patients on CT. Treatment response was noted in 12/17 patients (70.6%) on PET-CT. Eleven out of 17 (65%) patients showed response in both CT and PET-CT. Five out of 17 patients showed no response in both. Discordant findings on CT and PET were noted in one (5.9%) patient where PET showed response but no response was seen on CT. Two patients with initial response but with distant metastases expired during follow-up.

**Conclusion:**

PET-CT has potential in the initial staging of NB. PET-CT also appears to be a good modality for response assessment in patients with moderate and high FDG uptake on the baseline scan. However, no significant beneficial effect was seen in patients with low baseline FDG uptake.

## INTRODUCTION

Neuroblastoma (NB) is almost exclusively a pediatric neoplasm and the most common extracranial solid tumor in children, accounting for 8-10% of all childhood cancers.[1] It is the most common cancer diagnosed during infancy. Majority of children (62-70 %) have metastatic disease at the time of presentation.[1,2] Being a tumor of the neuroblasts of the sympathetic nervous system, the adrenal cells are the commonest site of origin (greater than 50%) followed by other retroperitoneal sites, mediastinum, pelvis and neck.

Various investigations are required for correct staging of the tumor. Conventional diagnostic imaging modalities include plain radiograph, ultrasound, contrast enhanced computerized tomography (CECT) and magnetic resonance imaging (MRI).[3,4] These imaging modalities, however, have their own limitations as they can only delineate the anatomy. Functional imaging modalities play a major role in staging. Bone scintigraphy is done commonly and helps detect cortical bone metastasis. However, pure lytic lesions can be missed on bone scan. The potential specificity and sensitivity of I-123/I-131 meta-iodobezylguanidine (MIBG) scintigraphy for evaluation of bone and soft tissue involvement by NB is attractive.[5,6] Catecholaminergic cells take up MIBG and hence the uptake in NB. The ability to screen the whole body in one non-invasive examination is a great advantage over other anatomy-based procedures. MIBG scan, however, has limitations as it may miss small lesions and may not be able to delineate the extent or localize the anatomic site. There is no single modality as of now which suffices for determining the extent of disease and assessing the response to therapy in these patients.[7]

Conventional imaging modalities wherein both the criteria described in literature, the World Health Organization (WHO) criteria and response evaluation criteria in solid tumors (RECIST), depend heavily on change in tumor size which would occur only if the chemotherapy is cytocidal.[8–10] The role of positron emission tomography-computed tomography (PET-CT) has been used for initial staging, treatment response evaluation and detection of recurrent cancer.[7] PET-CT has been shown to change the staging of various cancers. It also detects early changes in tumor physiology and biochemistry that result from efficacious therapy. Thereby, PET-CT overcomes the above-mentioned limitation of response evaluation based on reduction in size. There is little data published on PET-CT regarding initial staging of NB. In addition, no study has been done so far to assess the early response to neoadjuvant chemotherapy (NACT) in NB patients. We, therefore, planned this prospective study to investigate the role of PET-CT in initial staging and treatment response evaluation in NB.

## PATIENTS AND METHODS

### Patients

The study was performed according to the approved guidelines of the institutional ethical committee. Informed written consent was taken from parents of all patients before the study. A total of 17 patients of histologically confirmed new cases of NB with no prior treatment were prospectively included in the study. Patients in whom the tumor was amenable to upfront surgical resection, patients with poor general condition, patients who could not be sedated and patients where the parents refused to give consent were excluded from the study. There were 14 male and 3 female patients; age range 2-132 months (mean 51.5 months) [Table 1].

**Table 1:** Patient characteristics

Characteristics	Values
Number of patients enrolled	17
Age (in months)	
Mean	51.5
Range	2-132
Sex	
Male	14
Female	3
Total number of primary lesions	17
Site of lesions	
Suprarenal	11
Paraspinal	5
Cervical	1
Stage	
INSS-3	10
INSS-4	7

All patients were investigated as per revised international criteria for NB diagnosis, staging and response to treatment.[3,4] Twenty-four hour urinary catecholamines were used as tumor markers. Histological diagnosis was done by trucut biopsy or fine needle aspiration cytology. Contrast enhanced CT/MRI were used to determine the local extent of disease. Potential hematogenous and lymphatic metastatic sites were evaluated by CECT scan of the abdomen and chest or MRI, bone scan and bone marrow aspiration. Baseline evaluation was performed within 2 weeks preceding the start of treatment. In all patients, the same method of assessment was used to characterize the lesion at baseline and during follow-up. Patients were staged according to International NB Staging System (INSS).[3,4] A baseline whole body PET-CT scan was performed. Following the baseline scan, stage 1, 2A, 4s patients underwent two cycles of chemotherapy with cyclophosphamide and adriamycin. Cisplatinum and etoposide were additionally used in patients with stage 2B, 3, 4 disease. A second PET-CT scan was performed after two cycles of chemotherapy.

### PET-CT procedure

After fasting for at least 4 hrs and with patients in a resting state, in a quiet room, a dose of 5.3 MBq/kg (0.14 mCi/kg) of ^18^ F-flourodeoxyglucose (FDG) was injected intravenously. Older children were instructed to lie still whereas smaller children were encouraged to sleep. Sedation was done when needed using 0.1 mg/kg midazolam to avoid motion artifacts. PET-CT scan was acquired on a dedicated PET-CT scanner approximately 60 mins after intravenous injection of radiotracer. CT scan acquisition was performed on spiral dual slice CT with a slice thickness of 4 mm and a pitch of 1. Image was acquired using a matrix of 512×512 pixels and pixel size of about 1 mm. After the transmission scan, 3D PET acquisition was taken for 3-5 mins per bed position for one-two bed position. PET data was acquired using matrix of 128×128 pixels with a slice thickness of 1.5 mm. CT-based attenuation correction of the emission images were employed. PET images were reconstructed by iterative method ordered subset expectation maximization (two iterations and eight subsets) with a filter of 5 mm. The CT images were acquired and reconstructed using optimized parameters for attenuation correction. Data obtained from the CT acquisition was used for low noise attenuation correction of PET emission data and for fusion of attenuation corrected PET images with the corresponding CT images. After completion of PET acquisition, the reconstructed attenuation –corrected PET images, CT images and fused images of matching pairs of PET and CT images were reviewed in axial, coronal and sagittal planes and in maximum intensity projections, three-dimensional cine mode. PET images were assessed to identify areas of increased radiotracer uptake. Corresponding areas in the CT images and fused PET-CT images were corroborated.

### Data interpretation

Baseline FDG PET-CT study was performed for staging. Repeat PET-CT was done after two courses of NACT to evaluate treatment response. The PET-CT response was correlated with histopathology and clinical improvement. Histopathology was available in nine patients and remaining eight patients were evaluated based on clinical follow-up of minimum 6 months.

Qualitative criteria: FDG uptake in the lesion was compared with FDG uptake in normal liver parenchyma. Lesions were characterized as showing intense, moderate and mild FDG uptake when FDG uptake in tumor was significantly, moderately and slightly high, respectively, when compared with normal liver parenchyma. When uptake in lesion was equal or less in comparison with normal liver parenchyma then it was labeled as no FDG uptake. Where the liver had diffuse metastasis with no normal liver parenchyma visible on conventional imaging, normal splenic parenchyma was used as a comparison parameter.

Quantitative criteria: Standardized uptake value (SUV) in pediatric patients is not routinely being used in our institute. We, therefore, employed a method wherein we used the lesion to background (liver) ratio in the baseline as well as the scan done post two cycles of NACT. Comparison was made between the baseline scan and scan done post two cycles of NACT and the percentage improvement in the lesion to background SUV was calculated for all patients. As with other tumors, a 50% change in lesion to background ratio was taken as the cut-off value to classify as response.[11] In case of CT, RECIST criteria were used.[10]

## RESULTS

### PET-CT in staging

Out of the 17 patients, all 17 had undergone CECT to define the primary tumor and delineate the local extent of the disease. Primary tumor was seen in all patients on CECT (suprarenal 11, paraspinal 5, cervical 1). The size of the tumor ranged from 2.4 to 16.3 cm. Regional lymph nodes involvement were seen in 7/17 patients. A total of 69 lymph nodes were identified in seven patients. No other distant lesions were seen as the CT scan was limited to the primary site of disease.

Twelve of 17 patients underwent MIBG scan and bone scan (5/12 stage IV, 7/12 stage III). The remaining five patients could not undergo MIBG and bone scan due to technical limitations. MIBG uptake was seen in the primary lesion in 11/12 patients. The twelfth patient did not show any uptake. No lymph node could be identified distinct from the primary tumor in any of 12 patients who had MIBG scan. MIBG uptake in bones was seen in 3/12 patients. A total of 12 skeletal lesions were seen on MIBG scan. Bone can showed skeletal metastasis in 3/12 patients. A total of 12 skeletal lesions were identified on bone scan. All bone lesions were concordant on MIBG and bone scan.

**Figure 1:**
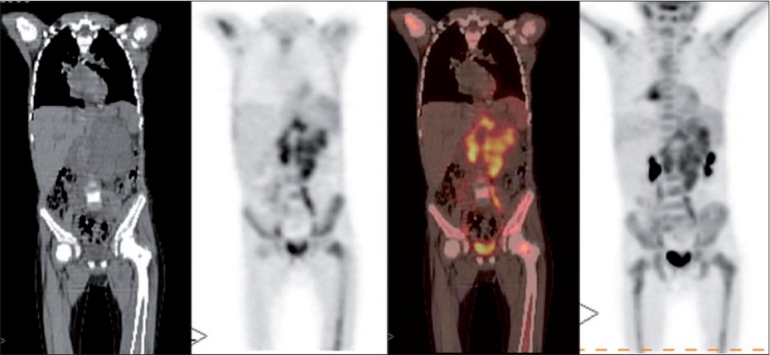
CT, PET, PET-CT and maximum intensity projection images (Left-Right) showing the presence of extensive disease in the abdomen and diffuse marrow metastasis

**Figure 2:**
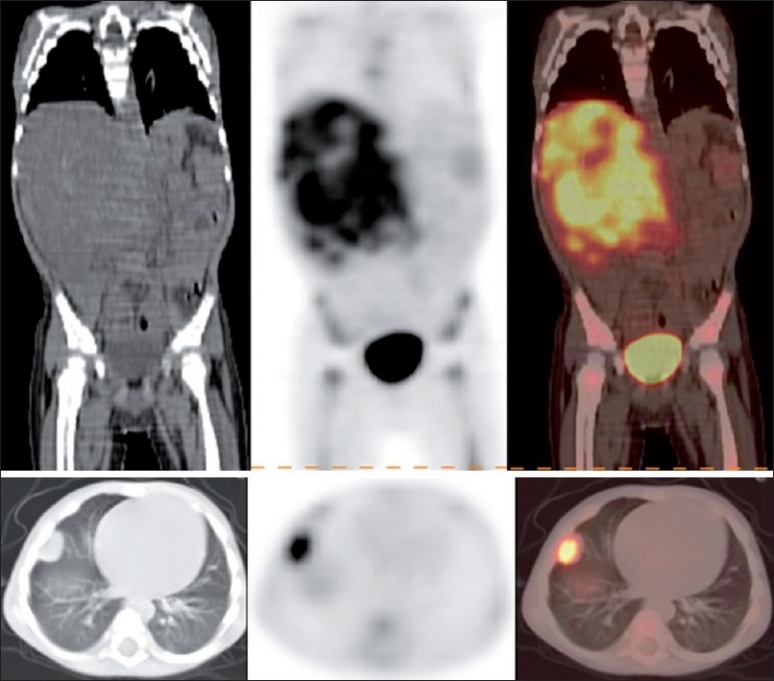
CT, PET and PET-CT images (L-R) showing the right suprarenal mass occupying right side of abdomen, pushing the liver superiorly and diffuse marrow metastasis (upper row). Transaxial CT, PET and PET-CT images in the same patient showing the presence of lung metastasis (lower row)

Baseline PET-CT showed increased FDG uptake in the primary lesion in all 17 patients. Intense uptake was noted in seven patients, moderate in four patients and mild in six patients. The size of the primary tumor on the baseline CT ranged from 2.9 to 16.6 cm (mean 7.3 cm) [Table 2]. The lesion to background SUVmax ratio in the baseline PET-CT ranged from 0.8 to 7.0 cm (mean 3.5 cm) [Table 2]. Lymph node metastasis was seen in 7/17 patients. Regional lymph nodes could not be counted due to the high uptake in the primary tumor. Nineteen lymph nodes other than the regional lymph nodes were identified. Enlarged cervical lymph nodes with increased FDG uptake were seen in one patient (other than those mentioned above) with suprarenal NB which on biopsy came out as benign. PET-CT detected metastasis to the bone marrow and lung in two patients and meningeal metastasis in 1 patient [Figures 1 and 2]. Four additional skeletal lesions were seen apart from those seen on MIBG and bone scan.

**Table 2:** Results

Age (months)	Sex	Stage	Size on ct			SUV lesion to background ratio			Response			Period of follow-up (months)	Current disease status
			Baseline (cm)	Post two cycles chemother-apy (cm)	% change	Baseline	Post two cycles chemother-apy	% improvement	CT	PET	Clinical		
72	M	4	6.5	3.6	45	7.0	1.5	79	+	+	CR	9	Died
108	M	3	4.8	4.8	-	1.2	1.2	-	-	-	PR	29	DF
20	M	4	10.6	5.8	45	6.0	1.2	80	+	+	PR	26	Died
2	M	3	7.8	7.1	9	1.3	1.2	8	-	-	CR	43	Died
36	M	3	10.8	10.1	11	6.8	1.1	84	-	+	CR	23	DF
72	M	4	6.7	3.0	55	5.0	1.5	70	+	+	PR	25	DF
18	F	3	2.9	-	100	1.5	-	100	+	+	CR	32	DF
108	M	3	16.6	14.1	15	0.8	0.8	-	-	-	PR	18	DF
132	M	3	12.6	8.4	33	1.6	0.2	88	+	+	CR	14	DF
2	M	3	4.2	3.5	17	1.8	1.0	45	-	-	CR	14	DF
6	F	3	3.4	1.6	53	4.0	1.7	58	+	-	PR	35	DF
72	M	4	11.0	6.8	48	2.6	1.6	61	+	-	PR	13	DF
24	F	4	10.3	10.2	1	4.2	6.0	-	-	-	PR	10	DF
48	M	3	4.5	-	100	3.2	-	100	+	+	CR	8	DF
120	M	4	6.0	3.2	47	5.4	2.1	61	+	+	PR	8	DF
24	M	4	10.8	-	100	4.6	-	100	+	+	PR	7	DF
12	M	3	4.5	-	100	2.4	-	100	+	+	CR	6	DF

### Comparative results of various modalities used for staging

The primary tumor was well-delineated in all patients on CECT and PET-CT. MIBG scan was performed in 12 patients. MIBG uptake was seen in 11/12 patients.

Regional lymph nodes were best identified on CECT scan. Owing to high radiotracer uptake in PET scan and MIBG scan, and also the lack of anatomical markers on MIBG scan, regional lymph nodes could not be identified separately from the primary tumor.

Distant metastasis could not be picked up on the baseline CECT scan as the CECT scan was limited to the site of the primary lesion. Bone scan could identify skeletal metastasis in 3/12 patients. MIBG scan showed skeletal metastasis at the same sites which were seen on bone scan. No additional sites of metastasis were seen. PET-CT detected additional bone marrow, lung and meningeal metastases, in addition to four more skeletal lesions.

### PET-CT in response evaluation

Response evaluation was done using PET-CT scan alone as all patients showed FDG uptake in the baseline PET-CT. No follow-up CECT, MIBG or bone scan was done for response evaluation. Non-contrast CT of PET-CT was used to measure the size of lesion whenever present to see the treatment response as per RECIST criteria.

### Follow-up scan (After two cycles of NACT)

The size of the primary tumor on the follow-up CT ranged from no mass to 14.1 cm (mean 5.7 cm) [Table 2]. The lesion to background SUVmax in the follow-up PET ranged from no uptake (as mass was completely resolved) to 6.0 (mean 1.2) [Table 2]. Reduction in the size of the primary lesion was seen in 11/17 patients. No change was seen in 6/17 patients. Reduction in the lesion to background SUVmax of the primary lesion was seen in 12/17 patients. No change was seen in 5/17 patients.

Complete resolution (CR) of lymph nodes and marrow metastasis were seen in 2/7 patients and of bone metastasis in one patient. CR of FDG uptake was also seen in the meningeal metastasis. Partial response (PR) was seen in the other lesions.

### Response analysis

As the number of patients in the study was small, the patients were grouped into responders and non-responders for analysis purpose. Responders comprised of those with CR and PR. The patients with stable disease and progression of disease were classified as non-responders.

**Figure 3:**
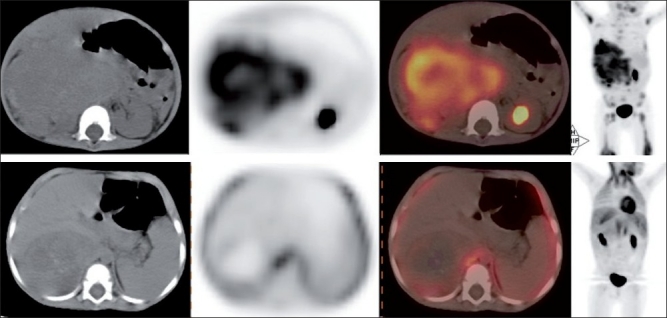
Transaxial CT, PET, PET-CT and maximum intensity projection images (L-R) images of a patient with NB. Baseline scan (upper row) shows a right suprarenal mass with intense uptake. Scan done post two cycles of NACT (lower row) shows reduction in the size of the mass as well as FDG uptake suggestive treatment response in CT and PET-CT

**Figure 4:**
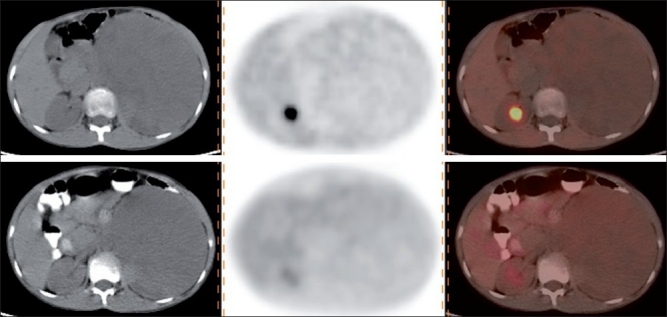
Transaxial CT, PET, and PET-CT images (L-R) images of a patient with NB. Baseline scan (upper row) shows a left suprarenal mass with mild uptake. Scan done post two cycles of NACT (lower row) shows no significant reduction in the size as well as FDG uptake. These finding are suggestive no treatment response on PET-CT and CT

**Figure 5:**
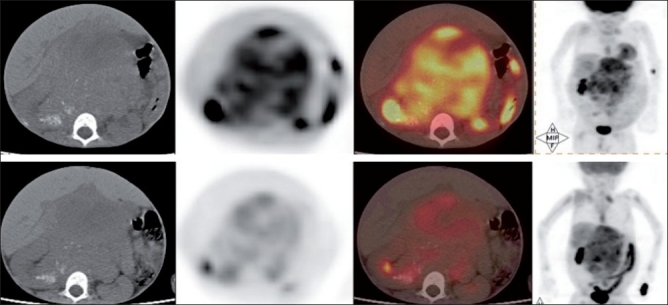
Transaxial CT, PET, PET-CT and maximum intensity projection images (L-R) images of a patient with NB. Baseline scan (upper row) shows a right suprarenal mass with intense uptake. Scan done post two cycles of NACT (lower row) shows no significant reduction in the size of the mass whereas there is a reduction in the FDG uptake. These finding are suggestive treatment response on PET-CT but no significant response on CT

**Figure 6:**
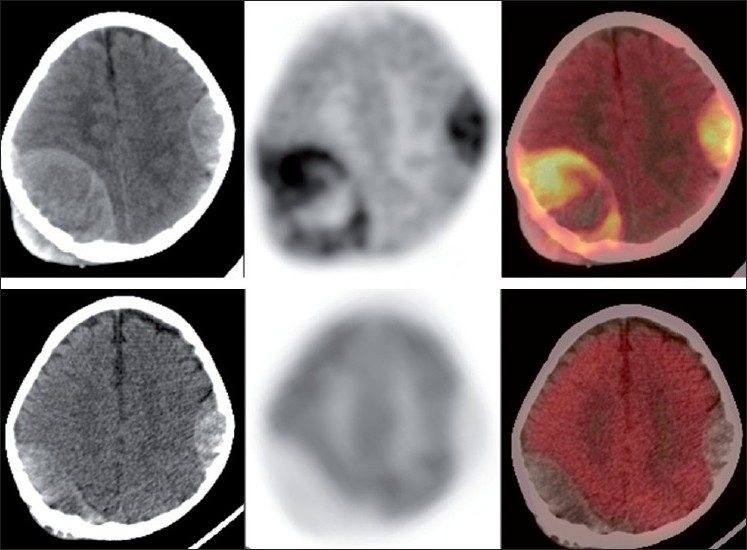
Transaxial CT, PET and PET-CT images (L-R) images of a patient with NB. Baseline scan (upper row) shows presence of meningeal metastasis. Scan done post two cycles of NACT (lower row) shows significant reduction in the FDG uptake. These finding are suggestive significant treatment response on PET-CT but partial significant response on CT

Eleven out of 17 (65%) patients showed response in both CT and PET-CT [Figure 3]. Five out of 17 patients showed no response in both [Figure 4]. Discordant findings on CT and PET were noted in one (5.9%) patient where PET showed response but no response was seen on CT [Figure 5]. Two patients with initial response but with distant metastases expired during follow-up [Figure 6].

A comparison between the pre NACT and post two cycles NACT scan is shown in Table 2. Clinically, all 17 patients were classified as responders (8 CR, 9 PR). Among the 8 CR, 6 were true positives and 2 were false positives. Among the 9 PR, 8 were true positives and 1 was false positive.

## DISCUSSION

Contrast enhanced CT is the most commonly used modality for the assessment of primary disease and lymph node involvement.[2,12,13] In our study too, CECT accurately delineated the site of the primary tumor and lymph node involvement. CECT was, however, not able to detect distant metastasis as it was confined to the site of the primary disease. Also, in recent years, MRI appears to be a more usefulmodality for the staging of NB and for visualization of anatomical details of the primary tumor, including relationships with the bloodvessels.[14,15] Sixty percent of patients of NB have metastasesin cortical bone, bone marrow, lymph nodes and liver.[16] Metastasis to the lung or brain, though rare, is also seen.[17,18] Forty-one percent of patients in our study had distant metastasis at the time of initial presentation which led to upgradation of the disease stage. These lesions would have been missed had whole body imaging not been performed. Most NB concentrate MIBG and is used routinely for the initial staging of the disease, evaluation of response to treatment, as well as the detection of recurrence. In 5-7% of cases, however, MIBG scintigraphy is negative at presentation.[19,20] MIBG scintigraphy has high sensitivity (93%) and specificity (100%) for thediagnosis of NB.[13,21] In the present study, out of 12 patients who underwent MIBG scintigraphy, 11 (92%) showed uptake in the primary tumor. False negative results are usually seen due to pharmacological interference, differentiation and maturation of tumor cellsand due to acquisition parameters such as low count rate and resolution constraints.[22,23] There was no history of drug intake in this one patient in our study. The reason for non-concentration of MIBG in this patient then being a combination of degree of differentiation and resolution constraints. MIBG scintigraphy was, however, not able to delineate regional lymph nodes separate from the primary tumor due to lack of anatomical details. Addition of SPECT and CT to MIBG scintigraphy can increase the accuracyof both methods.[24] False positives can result due to misinterpretation of physiological uptake and can be overcome by serial imaging over time an also by the addition of SPECT/CT. In patients where MIBG uptake is not seen, PET using^18^ F-FDG, ^18^F-dihydroxyphenylalanine (DOPA), or ^68^Ga-(DOTA-D-Phe[1]-Tyr[3]-octreotide)(DOTATOC)might be indicated.[25,26] Skeletal scintigraphy has long been used as the procedure of choice to assess bony involvement in diseases of diverse aetiology including NB.[27,28] There seems to be a complementary role for MIBG and MDP for evaluation of bone metastasis in patients with NB.[23,28,29] Three out of 12 patients in our study showed presence of skeletal metastasis on bone scan. All lesions were concordant on bone scan and MIBG scan. A study by Bouvier *et al*, comparing MIBG and bone scans, reported similar sensitivities (87.5%) for detection of skeletal metastasis. However, the specificity of MIBG was much higher (100%) as compared to bone scans (81%).[30]

PET-CT in recent years has emerged as an effective functional imaging modality for solid tumors such as NB. Earlier studies have shown that NB and their metastasis including those that did not absorb MIBG intensely accumulated FDG.[31] PET was also found to be equal or superior to MIBG for identifying NB in soft tissue and extracranial skeletal structures, for revealing small lesions and for delineating the extent and localizing sites of disease. PET and MIBG scans showed more skeletal lesions than bone scans, apart from those in the cranial vault.[8] In our study, all patients showed FDG uptake in the primary tumor, including the one which did not concentrate MIBG. However, due to the high FDG uptake in the primary tumor, regional lymph nodes could not be delineated separately. PET-CT detected additional skeletal lesions due to its better resolution capacity. Metastasis to the marrow, lung and meninges were missed on MIBG and bone scan and detected by PET-CT. Hence, PET-CT was beneficial in staging of NB as it has the added advantage of detecting the primary tumor, skeletal and soft tissue metastasis in a single investigation. An earlier study on a larger number of patients comparing the diagnostic utility of ^123^I-MIBG scintigraphy and ^18^F-FDG PET in NB, has shown that ^18^F-FDG PET was superior in depicting stage 1 and 2 NB. PET-CT was also useful in patients with tumors that either did not accumulate or poorly accumulated MIBG.[32]

Early chemotherapy response evaluation in pediatric tumors hasbeen widely studied in lymphomas.[33,34] As per our knowledge, there is no study, as of now, assessing early response to NACT in NB patients. We assessed the response to NACT after two cycles and observed that 65% of our patients showed improvement in both CT and PET-CT. All these patients had high uptake on the baseline scan. Histopathology was available in nine patients and revealed poorly differentiated tumor in five patients, undifferentiated in two patients and ganglioneuroblastoma with neuroblastic elements in another two patients. One patient showed discordant findings on PET and CT. CR of FDG uptake with no change in size was seen in this patient. This was because PET-CT being a metabolic imaging modality detected changes in the radiotracer uptake before structural alterations could take place. Five patients showed no response on PET as well as CT. Four out of these five patients had low baseline FDG uptake. All four patients are disease free on follow-up thus indicating lower efficacy of PET-CT for assessment of chemotherapy response in patients with initial low FDG uptake. Histopathology available in two of these patients with lower baseline uptake (patients 2 and 8) revealed ganglioneuroblastoma with neuroblastic elements. One patient had high baseline FDG uptake. PET-CT done post two cycles of NACT in this patient showed progression of the disease process.

In our study, seven out of 17 patients had symptomatic and PET findings mismatch. Out of the seven patients not showing any response on PET, three showed CR and four PR clinically. During follow-up, one of them died and the remaining six continue to be disease-free. Three patients expired during follow-up. In two of these patients, the primary tumor had shown good response to NACT clinically, on CT and PET-CT. One had shown response clinically; however, there was no response on CT/ PET-CT. Two of these patients had distant metastases at the time of diagnosis, thus indicating a worse prognosis irrespective of the FDG uptake and response in the primary tumor. The study, however, has its own limitations. The total number of cases is small. The follow-up period is also short in some patients so the real prognostic meaning of the degree of FDG uptake and long-term response to induction chemotherapy cannot be assessed. As non-contrast CT was used in PET-CT, characterization of structures was inferior and hence a separate contrast CT was required most of the times.

## CONCLUSION

PET-CT has potential in the initial staging of NB. PET-CT also appears to be a good modality for response assessment in patients with moderate and high FDG uptake on the baseline scan. However, no significant beneficial effect was seen in patients with low baseline FDG uptake.
